# Oleuropein Transcriptionally Primes *Lactobacillus plantarum* to Interact With Plant Hosts

**DOI:** 10.3389/fmicb.2019.02177

**Published:** 2019-09-18

**Authors:** Laura Santamaría, Inés Reverón, Laura Plaza-Vinuesa, Juan Carlos Oliveros, Blanca de las Rivas, Rosario Muñoz, Félix López de Felipe

**Affiliations:** ^1^Laboratorio de Biotecnología Bacteriana, Instituto de Ciencia y Tecnología de los Alimentos y Nutrición (ICTAN-CSIC), Madrid, Spain; ^2^National Center for Biotechnology (CNB-CSIC), Madrid, Spain

**Keywords:** oleuropein, transcriptomics, *Lactobacillus plantarum*, signaling, quorum sensing, inmunomodulators

## Abstract

Oleuropein (OLE) is a secoiridoid unique to *Oleaceae* known to play a role in the plant–herbivore interaction. However, it is not clear how this molecule is induced to mediate plant responses to microbes and how microbes, in turn, withstand with OLE. To better understand how OLE affects the plant–microbe interaction, the contribution of differential gene expression in the adaptation to OLE was characterized by whole genome transcriptional profiling in *Lactobacillus plantarum*, a bacterium associated to the olive. OLE downregulated functions associated to rapid growth, remodeled membrane phospholipid biosynthesis pathways and markedly repressed the expression of several ABC transporters from *L. plantarum*. Genes encoding the plantaricin and *lamABDCA* quorum-sensing (QS) systems were down-regulated indicating the potential of OLE as a QS-antagonist. Notably, OLE diminished the expression of a set of genes encoding inmunomodulatory components and reoriented metabolic pathways to increase protein acetylation, probably to attenuate plant immunity. Responses were also triggered to repress the transport of acetoin and to buffer reactive oxygen species accumulation, two signals involved in plant development. The results suggest that OLE could act as a signaling molecule in the plant–microbe interaction and facilitate the accommodation of beneficial microbes such as *L. plantarum* by the plant host, via controlled expression of bacterial molecular players involved in this reciprocal interplay.

## Introduction

Oleuropein (OLE) is the most abundant among a set of secoiridoids that are unique to *Oleaceae* (Amiot et al., 1986). This phenolic compound is found in almost all parts of the *Olea europaea* tree including seeds, roots or bark, is the major phenolic compound in stem tissues (Báidez et al., 2007), and it is abundant in leaves and olive fruits (Hashmi et al., 2015). OLE is known to play a role in the *Oleaceae* plant–herbivore interaction acting as precursor of alkylators that are toxic to generalist herbivores but not to hervibores specialized in feeding on iridoid-containing plants (Konno et al., 1999). However, up to the best of our knowledge there is no evidence that OLE can play a similar shaping role in the plant-microbe interaction to influence the composition of the phytomicrobiome associated to *Oleaceae*.

In the context of the intimate interaction with their hosts, members of plant microbiomes can induce defense phytohormones when recognized by the plant. These phytohormones translate this perception, via a complex signaling network, into an effective immune response (Hacquard et al., 2017). In turn, both pathogenic and beneficial microbes have evolved different strategies to modulate the plant immune responses (Hacquard et al., 2017). This reciprocal interaction between the plant and members of its associated phytomicrobiome is conducted via signaling molecules (Smith et al., 2015). A known example is the signaling between leguminous plants and rhizobial cells involving plant isoflavonoids that chemoattract rhizobial cells to the roots and activate bacterial key genes coding for lipo-chitooligosaccharides which in turn signal and stimulate plant growth (Hassan and Mathesius, 2012; Oldroyd, 2013). Communication between members of the phytomicrobiome is also conducted via signaling (Smith et al., 2015). Interestingly, some quorum sensing (QS)-signals, a sort of signals used for communication between bacteria, have been shown to modulate plant-microbe interactions by eliciting immune responses and provoke plant growth responses (Hartmann et al., 2014). Curiously, components of two QS-systems involved in the production of a new cyclic thiolactone (Sturme et al., 2005) or the bacteriocin plantaricin (Diep et al., 2003) from *Lactobacillus plantarum* (a bacterium associated to the olive), have been also shown to act as signals eliciting immunomodulatory responses (Meijerink et al., 2010; van Hemmert et al., 2010). In other cases, the microbial product can act directly as signaling molecule to increase plant growth as it occurs for Thuricin 17, a bacteriocin produced by a plant growth promoting strain of *Bacillus thuringiensis* (Gray et al., 2006).

Recently the olive orchard microbiome has been deciphered (Fausto et al., 2018) and lactic acid bacteria, including *Lactobacillus*, were among the dominant genera in the three compartments studied (soil, leaves and xylem sap). These results are in line with the finding that the *Lactobacillales* order is one of the bacterial endophytes most abundant that colonize the Mediterranean olive trees (Müller et al., 2015), and also with the presence of lactic acid bacteria among the phytomicrobiomes of many plants (Lamont et al., 2017; Pontonio et al., 2018). At the species level *L. plantarum* and *Lactobacillus sakei* have been associated to the olive rizhosphere (Fhoula et al., 2013). The membership of *Lactobacillus* in the olive microbiome and its presence in the olive endosphere points toward a close association, albeit the exact role performed by these microorganisms in the olive-phytomicrobiome community has not been yet deciphered. In addition, the signaling systems that influence the microbial diversity and regulate the reciprocal dialog between the olive and its phytomicrobiome are not currently identified. OLE could be a candidate plant signal to regulate this interaction as it is widespread in the olive (Hashmi et al., 2015), is the majority phenolic compound in, at least, olive tissues such as the xylem and phloem (Báidez et al., 2007) and displays an uneven *in vitro* impact on microorganisms associated to the olive. For example, OLE is inhibitory *in vitro* to the pathogenic fungus *Verticillium dahliae* Kleb. (Báidez et al., 2007), however, it was not found inhibitory against *L. plantarum* in three *in vitro* independent studies (Rozès and Peres, 1996; Marsilio and Lanza, 1998; Landete et al., 2008). When compared to other nine phenolic compounds OLE was, besides tyrosol, the sole phenolic that did not inhibit *L. plantarum* even at concentrations that vastly exceed the OLE concentrations found in olive (Landete et al., 2008). Altogether, these results provide a hint that this secoiridoid could be involved in shaping the phytomicrobiome associated to *Oleaceae*.

Among lactobacilli, *L. plantarum* is part of the indigenous olive fruit microbiota (Landete et al., 2010; Ruiz-Barba and Jiménez-Díaz, 2012), present in the rizhosphere of olive trees (Fhoula et al., 2013), found to display high tolerance *in vitro* to OLE (Rozès and Peres, 1996; Marsilio and Lanza, 1998; Landete et al., 2008) and used as a model to study the molecular responses to other types of plant phenolic compounds (Curiel et al., 2011; Reverón et al., 2012, 2013, 2015, 2017, 2018). Therefore this bacterium has been chosen in this study as a suitable microbe to shed light on the potential role of OLE as signaling molecule in the reciprocal communication between *Oleaceae* and members of its associated phytomicrobiome. To this goal, *L. plantarum* WCFS1 was exposed to OLE in order to determine the OLE-responsive genes and gene circuits involved in the global transcriptome responses to this secoiridoid by using DNA microarrays.

## Materials and Methods

### Bacterial Strain, Culture Conditions

The model strain *Lactobacillus plantarum* WCFS1 was kindly provided by Dr. Michiel Kleerebezem (NIZO Food Research, Netherlands). The genome of this strain was the first published among *Lactobacillus* species and it has a large size of 3.3 M thought to be related to the diversity of environmental niches in which *L. plantarum* spp. is encountered. Due to the early publishing of its genome sequence this strain has been subject of extensive functional genomic research (van den Nieuwboer et al., 2016). Based on this knowledge and because it was previously used as model strain to study the molecular responses to treatments with several classes of plant phenolic compounds (see section Introduction) and olive-derived raw materials such as olive oil (Esteban-Torres et al., 2017), *L. plantarum* WCFS1 was chosen for this study. *L. plantarum* WCFS1 was grown in Man-Rogosa-Sharpe (MRS) broth (Difco Laboratories, Madrid, Spain) at 30°C without shaking.

### RNA Extraction

For RNA isolation twelve paired independent *L. plantarum* WCFS1 batch cultures (50 mL each) were grown in MRS to an OD_600_ ≈ 0.8–0.9. Then, OLE (15 mM final concentration) (Extrasynthèse, Genay, France) was added to a culture of each pair (12 cultures). After 10 min, the cells exposed to OLE and their respective non-induced controls were centrifuged at 4°C. The pellet was mixed with 2 mL of quenching buffer (60% methanol, 66.7 mM HEPES, pH 6.5, −40°C). Following quenching, the cells were centrifuged at 9,000 × *g* for 10 min at −10°C and suspended in an extraction mixture (500 mL 1:4 chloroform-acid phenol, 30 mL of 10% SDS, 30 mL Na-acetate 3M pH 5.2, 400 mL Tris-EDTA buffer [10 mM Tris(hydroxymethyl)amino methane, 1 mM EDTA] pH 7.4, 15 mg of polyvinylpolypyrrolidone, and 500 mg of glass beads (ϕ, 75–150 mm). The cells were broken under frozen conditions using a cell disruptor (FastPrep^TM^ FP120. Bio101. Savant). Three cycles of 40 s were applied operating at 5 m/s speed with 1 min interval in ice between cycles. The suspension was then centrifuged at 4°C at 10,000 × *g* for 2 min. After two extractions with 500 mL of chloroform the supernatant containing the RNA was immediately frozen in liquid nitrogen, and stored at −80°C (Saulnier et al., 2007). The NanoDrop ND1000 instrument was used for RNA quantification. The A_260_/A_280_ and A_260_/A_230_ ratios were measured to check RNA purity. Integrity and quality of RNA samples were determined by electrophoresis on agarose gels. Two treatments with DNase I (Ambion) were applied and the absence of genomic DNA was confirmed by PCR (Saulnier et al., 2007).

### Microarray: cDNA Synthesis, Purification and Hybridization

Before first-strand cDNA synthesis, RNA integrity was evaluated using the Agilent 2100 Bioanalyzer (Agilent Technologies). Fluorescently labeled cDNA was obtained by using the SuperScript Indirect cDNA Labeling System (Invitrogen). The Cy3 and HyPer5 fluorescent dyes (Amersham Biosciences) were coupled to the aminoallyl-modified first-strand cDNA, and purification of probes was carried out with the CyScribe GFX Purification Kit. Labeling efficiency was assessed using the NanoDrop ND1000 spectrophotometer. Preparation of probes and hybridization at 65°C during 17 h was performed as described in the Two-Color Microarray Based Gene Expression Analysis Manual (Quick Amp Labeling) with Tecan HS Pro Hybridization (V. 5.7/Agilent Technologies). Slide *L. plantarum* WCFS1 8 × 15K microarray GE Agilent G2509F Oligo Microarrays (No. 026636) was custom designed and contains 60-mer probes that were based on the gene expression omnibus database (GEO Accession No.GPL5874). The oligo-microarray contained an average of three probes per transcript.

### Real-Time Quantitative RT-PCR Assays (RT-qPCR)

Real-time RT-qPCR was used to validate the microarray data. Amplification was carried out using a 7500 Fast System (Applied Biosystems). RNA was reverse transcribed using High Capacity cDNA Reverse Transcription Kits (Applied Biosystems). The specific primers used for the RT-qPCR assays were designed with the Primer Express 3.0 software and listed in the Supplementary Table S1. The SYBR Green method was used and each assay was performed in triplicate using SYBR Green real-time PCR Master Mix (Applied Biosystems). Amplification was initiated at 95°C for 10 min, followed by 40 cycles of 95°C for 15 s and 60°C for 1 min. Control PCRs were included to confirm the absence of primer dimer formation (no-template control), and to verify that there was no DNA contamination (without RT enzyme negative control). All real-time PCR assays amplified a single product as determined by melting curve analysis and by electrophoresis. A standard curve was plotted with cycle threshold (Ct) values obtained from amplification of known quantities of cDNAs and used to determine the efficiency (E) as E = 10^–1/slope^. The expression levels of target genes were normalized. The Bestkeeper analysis (Pfaffl et al., 2004) was applied, and the geometric mean of the most stably expressed housekeeping genes (16S rRNA, *gapB, dnaG*, and *gyrA*) was used as a normalization factor. The expression ratios measured by microarrays and by RT-qPCR assay were plotted, and the linear correlation coefficient was calculated (*y* = 0.9507x + 0.1473; *R*^2^ = 0.9758).

### Data Analysis

Images were captured with a GenePix 4000B (Axon) and spots quantified using GenPix software (Axon). Background correction and normalization of expression data were performed using the methods normexp and loess in LIMMA, respectively (Smyth and Speed, 2003). The expected False Discovery Rate (FDR) was controlled to be less than 5%. Genes were considered as differentially expressed when nominal *p-*values were <0.05 and had a fold change (FC) equal or higher than ±1.5. FC was calculated as the average of the FC between significantly regulated probes. Hybridizations and statistical analysis were performed by the Genomics Facility at National Center for Biotechnology, CSIC, Spain.

Finally, analysis of Gene Ontology (GO) terms and KEGG pathways associated to differentially expressed genes, was performed using DAVID tool (Huang et al., 2009). In the case of Gene Ontology terms, “GO Direct” subset was used. Terms with *p-values* less than 0.1 were considered as significant.

### Accession Numbers

The microarray data provided in this study have been deposited in NCBI Gene Expression Omnibus (Edgar et al., 2002) genomics data repository and are accessible through GEO Series accession number GSE90719.

## Results

### Global Analysis of the Transcriptomic Response to Oleuropein

**FIGURE 1:**
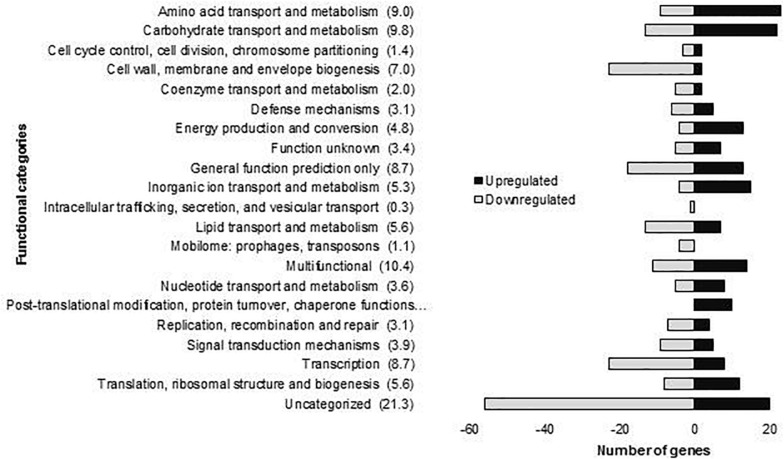
Number of *L. plantarum* WCFS1 genes by functional categories that were upregulated and downregulated in the presence of 15 mM oleuropein. The number in parentheses represents the percentage of differentially expressed genes in each category.

The molecular responses of *L. plantarum* WCFS1 after 10 min of OLE stress were determined by global transcriptome profiling using DNA microarrays. The short exposure time to OLE was chosen because we were interested in the very first transcriptional responses which can be masked by secondary effects activated with increasing exposure time. The OLE concentration used [15 mM (0.8%)] is similar to some of the amounts detected in several parts of the olive such as 0.3–0.5% found in olive tissues (cortex + phloem or xylem + pith, respectively) (Báidez et al., 2007), 0.7% in leaves (Tayoub et al., 2012) or 0.4% in olive fruit (Ruíz-Barba et al., 1993). The analysis showed 358 DE genes after 10 min of exposure to OLE, as compared to control conditions (*t* = 0). The DE genes were functionally classified according to their Cluster Orthologous Groups (COGs) categories (Figure 1). From these genes, which are listed in Supplementary Table S2, 155 were upregulated and 203 downregulated.

**FIGURE 2:**
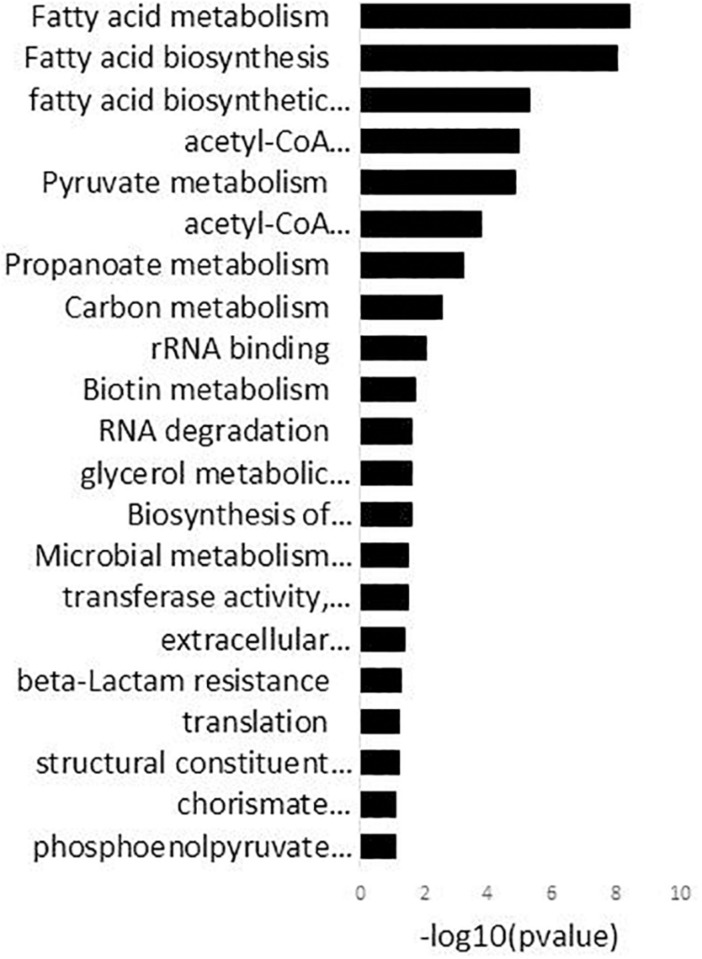
DAVID analysis of functional gene ontology (GO) categories and KEGG pathways related to the genes differentially expressed in 15 mM oleuropein. Fold enrichment (bars) parameter measures the magnitude of enrichment, both for up and down-regulated gene groups.

The enriched GO-DIRECT categories and KEGG pathways resulting from the functional analysis with DAVID (Figure 2) are described in more detail below.

### Oleuropein as Anti-quorum Sensing Metabolite: Reduced Transcription of the Plantaricin and the *lamBDCA* Quorum-Sensing Systems

The presence of OLE greatly affected the expression of two quorum sensing (QS) systems, one required for the production of plantaricin and other required for the production of LamD558, a cyclic thiolactone autoinducing peptide encoded by the *lamBDCA* operon (Sturme et al., 2005).

In *L. plantarum* WCFS1 the enzymatic machinery required for plantaricin production is encoded by the *pln* locus, which contains five operons (Diep et al., 2003). The most downregulated operon by OLE within the *pln* locus was *plnABCD* (*plnA* or *lp_0415*, *plnB* or *lp_0416*, *plnC* or *lp_0417*, *plnD* or *lp_0418*) (Supplementary Table S2). This operon codes for the known quorum sensing QS-system required for plantaricin A production. Plantaricin A, in turn, controls the expression of all the five operons from the *pln* locus (Diep et al., 2003). Accordingly, genes encompassed within the *plnEFI* (*plnE* or *lp_0422*, *plnF* or *lp_0421*, *plnI* or *lp_0419*), *plnJKLR* (*plnJ* or *lp_0406*, *plnK* or *lp_0405*, *plnL* or *lp_0404*) operons, which respectively code the EF and JK plantaricins; *plnGHSTUVWXY* (*plnG* or *lp_0423, plnH* or *lp_0424, plnV* or *lp_0428*) which codes for the ABC transporter and accessory proteins required to transport and secrete the peptides; and *plnMNOP* (*plnP* or *lp_0412*) coding for proteins of unknown function, were downregulated (Supplementary Table S2).

As to the LamBDCA QS-system, OLE provoked a marked downregulation of *lamC* or *lp_3581* (up to 3-fold) coding for the sensor histidine protein kinase (HPK) and *lamD* or *lp_3581a* (up to 4.6-fold) coding for the precursor auto-inducer peptide (AIP) of this system (Supplementary Table S2).

### Oleuropein Downregulates Genetic Loci With Immunomodulatory Capacities

Notably, the *lamBDCA* and some components of the *pln* (*plnEFI* and *plnGHSTUVWXY* operons) QS-systems that are downregulated by OLE have been reported to be involved in immunomodulation (Meijerink et al., 2010; van Hemmert et al., 2010). Interestingly, other *L. plantarum* loci with confirmed immunomodulatory capacities such as components of the *pts19ADCBR* locus (*lp_2647*, *lp_2650*) the prophage P2b protein 21 (*lp_2460*) (van Hemmert et al., 2010) and components of the *N*-glucosamine/galactosamine phosphotransferase system (*lp_2647* and *lp_2650*) (van Hemmert et al., 2010), were also down-regulated by OLE (Supplementary Table S2).

### Oleuropein Reduces the Expression of All the Four Capsular Polysaccharide Biosynthesis Gene Clusters of *L. plantarum*

Genes associated with the GO terms “extracellular polysaccharide biosynthetic process” and “transferase activity, transferring glycosyl groups” were significantly enriched in the DAVID analysis (Figure 2 and Supplementary Table S2). These genes, which were all downregulated, are encompassed within the four different surface polysaccharide gene clusters contained in the *L. plantarum* genome, *cps1* (*cps1D* or *lp_1180*, *cps1H* or *lp_1184*, *cps1I* or *lp_1185*), *cps2* (*cps2A* or *lp_1197*, *cps2B* or *lp_1198*, *cps2E* or *lp_1201*, *cps2F* or *lp_1202*, *cps2G* or *lp_1203*, *cps2K* or *lp_1207*), *cps3* (*cps3A* or *lp_1215*, *cps3F* or *lp_1222*, *cps3G* or *lp_1224*) and *cps4* (*cps4J*, *cps4I*, *cps4H*, *cps4G*, *cps4F*, *cps4E* or *lp_2099* to *lp_2104*) and *cps4* (*cps4C, cps4B, cps4A* or *lp_2106* to *lp_2108*). These genes code for proteins involved in different steps of repeating unit synthesis, export and polymerization of polysaccharides.

### Carbohydrate Uptake and Metabolism Functions Are Significantly Enriched in the Oleuropein-Responsive Transcriptome

Genes encompassed within the GO term “pyruvate metabolism” were also significantly enriched in the DAVID analysis (Figure 2). This response included the induction of the pyruvate oxidase (*lp_3589*), which produces acetyl-P from pyruvate and the pyruvate dehydrogenase complex (*lp_2151, lp_2153, lp_2154*) which forms acetyl-CoA (the precursor of acetyl-P) from pyruvate. In addition, genes coding for enzymes of the acetyl-CoA complex (*accB2* or *lp_1676, accC2* or *lp_1678, accD2* or *lp_1679*), which use acetyl-CoA to initiate the fatty acid biosynthesis (fab), and the acetate kinase (*lp_0210*) which forms acetate from acetyl-P, were downregulated.

The DAVID analysis enriched genes within the GO term “glycerol metabolic processes” (Figure 2). The dihydroxyacetone (DHA) transporter (*lp_0171*) was upregulated whereas the DHA phosphotransferase encoding genes (*lp_0168*, *lp_0169*) were downregulated. In addition, the glycerol-3-phosphate dehydrogenase (*glpD* or *lp_0371*) and the glycerol kinase (*glpK* or *lp_0370*) were up-regulated (Supplementary Table S2).

The “phosphoenolpyruvate-dependent sugar phospho transferase system (PTS) transporter” (Figure 2) was one of the largest categories significantly enriched by OLE in the transcriptome of *L. plantarum*. Genes encoding PTS transporters required for the uptake of mannose (*pts10B* or *lp_0587*), the sugar alcohol mannitol (*pts2CB* or *lp_0230*), threalose (*pts5ABC* or *lp_0265*), cellobiose (*pts6C* or *lp_0286*, *pts20A* or *lp_2780*, *pts20B* or *lp_2781*) and fructose (*pts16ABC* or *lp_2097*) were all induced in presence of OLE. In addition, the maltose proton transporter (*malT* or *lp_1729*) was also up-regulated. In contrast, two PTS systems involved in the transport of presently not known β-glucosides (*pts15A* or *lp_1398*, *pts30BCA* or *lp_3513*) and PTS systems for the transport of *N*-acetyl-glucosamine (GlcNAc) (*pts18CBA* or *lp_2531*), *N*-acetyl-galactosamine (GalNAc) (*pts19B* or *lp_2650*) and GlcNAc/GalNAc (*pts19A* or *lp_2647*) were downregulated.

### Oleuropein Affects Biological Functions Involved in Cell Proliferation

The DAVID analysis also enriched genes encompassed within several categories related to cell proliferation and stress. For example, strong downregulation was observed for up to 11 clustered genes coding for proteins involved in the initiation (*fabZ1* or *lp_1670*, *fabD* or *lp_1673*, *accB2* or *lp_1676*, *accC2* or *lp_1678*, *accD2* or *lp_1679*) and elongation (*fabH2* or *lp_1670*, *fabG1* or *lp_1674*, *fabF* or *lp_1675*, *fabZ2* or *lp_1677*, *fabI* or *lp_1681*) steps of the KEGG pathway “fatty acid biosynthesis” (FAB) (Figure 2) as well as the gene coding for the acyl carrier protein (*lp_1672*) (Supplementary Table S2). Other enriched genes involved in cell proliferative processes were those coding for ribosomal proteins encompassed within the GO term “structural constituent of ribosome” (Figure 2). Nine of these genes coding for the corresponding ribosomal proteins (*lp_0009* [RPLS6], *lp_1033* [RPL3], *lp_1034* [RPL4], *lp_1035* [RPL23], *lp_1036* [RPL2], *lp_1038* [RPLS19], *lp_1039* [RPL22], *lp_1045* [RPL14], *lp_1053* [RPS5]), were down-regulated (Supplementary Table S2). In addition, genes required to counter stress that code for molecular chaperones (*groES* or *lp_0727*, *groEL* or *lp_0728*) and alkaline (*asp1* or *lp_0929*) or cold shock (*lp_0997*) proteins, were induced by OLE. Some genes known for its role in countering oxidative stress such as *npr2* or *lp_2544* (NADH-peroxidase), *gshR2* or *lp_1253* (glutathione reductase), *msrA4* or *lp_1979* (methionine sulfoxide reductase) were up-regulated or down-regulated (*lexA* or *lp_2063*, transcriptional repressor of the SOS regulon). This response indicates that OLE induces anti-oxidant enzymes/proteins.

### Regulation of ABC Transporters by Oleuropein

The expression of a number of ABC transporters with different functions were differently regulated by OLE (Supplementary Table S2). Some of these transporters have been previously shown to be involved in the response to other classes of phenolic compounds. For example, the expression of the ABC transporter encoded by *lp_2739* and *lp_2740* genes, which is strongly downregulated by OLE (up to 20-fold), is also highly responsive to resveratrol (Reverón et al., 2018), *p*-coumaric acid (Reverón et al., 2012) and gallic acid (Reverón et al., 2015). In the same vein, genes in a locus (*lp_2741* to *lp_2744*) encoding an ABC transporter responsive to gallic acid (Reverón et al., 2015), were also downregulated by OLE. Besides modulation of these transporters, OLE affected the expression of ABC transporters with other functions. Among these are the induced genes coding for subunits of the ATP-dependent ABC oligopeptide transporter Opp (*oppABCDF* or *lp_1261* to or *lp_1265*). Interestingly, genes coding for an acetoin ABC transporter (*lp_0325*, *lp_0326*, *lp_0327*), were down-regulated.

## Discussion

**FIGURE 3:**
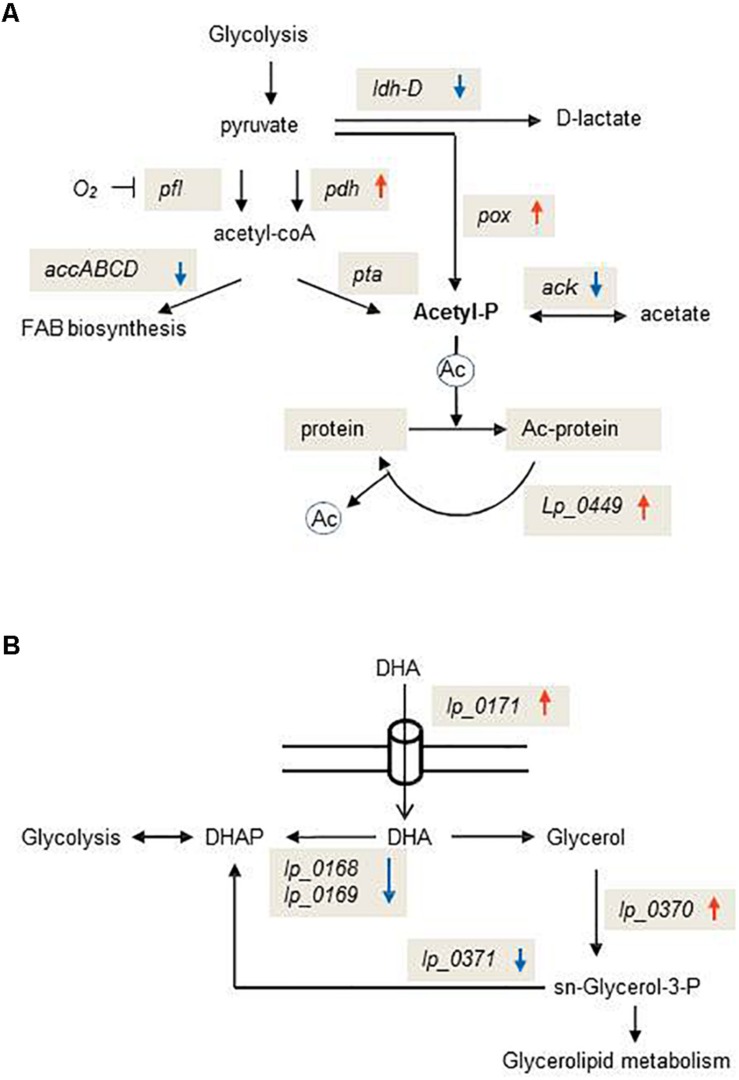
Schematic representation of exemplary metabolic pathways transcriptionally affected by oleuropein in *Lactobacillus plantarum* WCFS1. **(A)** Schematic diagram of the pathway for glycolytic production of acetyl-P and post-translational modifications in which this metabolite is involved. **(B)** Schematic diagram of the pathway for the production of the glycerolipid precursor, sn-glycerol-3-P. PDHc, pyruvate dehydrogenase complex; PFL, pyruvate formate lyase; AccABCD, acetyl-CoA carboxylase; LDH-D, D-lactate dehydrogenase; POX, pyruvate oxidase; ACK, acetate kinase; PTA, phospho-transacetylase. Ac-protein, acetylated protein. DHA, dihydroxyacetone; DHAP, dihydroxyacetone-phosphate. Upward red arrows indicate up-regulated genes; downward blue arrows indicate down-regulated genes.

Whole genome transcriptional profiling showed how *L. plantarum* WCFS1 responded to OLE. The expression of rRNA and *fab* genes, two major groups of genes associated to rapid growth, was markedly reduced in presence of this compound. *L. plantarum* strongly downregulated several ABC transporters whose expression was shown to be highly responsive to resveratrol (Reverón et al., 2018), *p*-coumaric acid (Reverón et al., 2012) and gallic acid (Reverón et al., 2015). This profile indicates that transport control across the membrane is a crucial strategy to hinder accumulation of OLE inside the cell. Accordingly, other plant signaling molecules such as jasmonate or salicylic acid can activate the expression of specific and non-specific multidrug efflux pump-encoding genes in plant pathogens (Ravirala et al., 2007; Antunez-Lamas et al., 2009) or symbionts such as *L. plantarum* (Reverón et al., 2012, 2018), leading to enhanced bacterial survival in the plant. Membrane phospholipid remodeling would be another mechanism to counter the effects of OLE, as a set of genes were transcriptionally modulated to increase the synthesis of sn-glycerol-3-P (obligated precursor of glycerophospholipids) from glycerol or DHA (Figure 3). In this regard, membrane phospholipid alterations have been recently reported to determine the bacterial adaptability to environmental stresses (Rowlett et al., 2017).

In bacteria, most of the global protein acetylation is dependent on acetyl-P formation and control of acetylation levels requires the involvement of a sirtuin class deacetylase (CobB) (Weinert et al., 2013). Our data indicate that OLE promotes protein acetylation in *L. plantarum* as this secoiridoid reoriented metabolic pathways toward acetyl-P accumulation (Figure 3) and induced the *L. plantarum* homologue of CobB (*lp_0449*) (Supplementary Table S2). Acetylation of plant proteins, which can be induced by molecules such as jasmonic acid, ethylene or salicylic acid (JA/ET/SA), plays a critical role to trigger appropriate immune responses during plant-pathogen interactions (Song and Walley, 2016). The effects of OLE on the *L. plantarum* transcriptome suggest that plant molecules can not only promote acetylation in plant histone and non-histone proteins (Song and Walley, 2016), but also in the microbes that interact with the plant host. Further studies will be necessary to understand if this post-translational modification in *L. plantarum* has consequences in triggering appropriate immune responses during *L. plantarum*-plant host interaction.

QS-signals are important modulators of microbe-plant interaction (Hartmann et al., 2014) and plants can perceive these signals and respond in different ways to alter QS impact (Bauer and Mathesius, 2004; Hartmann et al., 2014). Down-regulation of the plantaricin and the *lamBDCA* QS-systems shows the capacity of OLE to act as QS antagonist at the transcriptional level. Interestingly, some of the components of the plantaricin and LamBDCA QS-systems are involved in immunomodulation and regulate the cytokine output in immunomodulatory cells (Meijerink et al., 2010; van Hemmert et al., 2010). To trigger such immune responses these QS-components should have structures or chemical patterns unique to microbes that can be perceived as non-self/foreign by specific receptors of innate immune systems of both, vertebrates or plants (Boller and Felix, 2009). Therefore, it is likely that these QS-components are recognized by specific Pattern Recognition Receptors (PRRs) in plants. Supporting this hypothesis some QS-signals have been suggested as potential Microbe-Associated Molecular Patterns (MAMPs) in plants (Boller and Felix, 2009) and reported to induce defense responses in tomato (Schuhegger et al., 2006).

Interestingly, OLE not only downregulated these immunomodulatory QS-components but also other genes involved in immunomodulation which are encompassed in two loci encoding the *N*-acetyl-glucosamine/galactosamine phosphotransferase system and the prophage P2b protein 21 (Meijerink et al., 2010; van Hemmert et al., 2010). Since *L. plantarum* mutants in these genes and in components of the plantaricin and LamBDCA QS-systems increase the anti-inflammatory output of immune cells (Meijerink et al., 2010; van Hemmert et al., 2010), it is possible that transcriptional downregulation of these gene set by OLE could serve to dampen plant host immunity. It is to note that attenuation of plant immune responses by microbes is required for plant-microbe interactions and provide adaptation to the endophytic lifestyle (Hacquard et al., 2017). Other *L. plantarum* WCFS1 components involved in immunomodulation are the four polysaccharides (CPSs) synthesized by this bacterium. Deletion mutants in the four clusters of *L. plantarum* WCFS1 have been shown to alter immunomodulation by increasing the exposure of MAMPs to their receptors to induce signaling cascades (Remus et al., 2012; Lee et al., 2016). Based on the observed profile, it can be inferred that downregulation of the four *cps* clusters by OLE may facilitate the exposure of certain bacterial immunomodulatory components that contribute to instruct the plant immune system.

OLE induced several PTS transporters two of which were dedicated to the transport of mannitol or fructose, two out of the five main soluble components in olive source and sink tissues (Marsilio et al., 2001). This profile could be expected from a close association between *L. plantarum* and the olive as sugar transfer from the plant to the microbial symbiont is essential for symbiotic relationship (Pfister et al., 2017).

A set of genes involved in countering oxidative stress were induced by OLE. Reactive oxygen species (ROS) increase during the course of many biotic and abiotic stress conditions potentially resulting in damage of macromolecules. Interestingly, ROS have signaling roles in plant adaptation to stress, however, their levels have to be moderated during plant adaptation to act in signaling and not to be channeled to damage (Krishnamurthy and Rathinasabapathi, 2013). The observed induction by OLE of enzymes dedicated to counter oxidative stress may help to buffer ROS accumulation and facilitate its signaling role for plant adaptation to stress.

The oligopeptide ABC transporter was upregulated by OLE but induction of the involved *opp* genes was not accompanied by induction of endopeptidases (required to digest the oligopeptides for nutrition) or muropeptidases (needed for muropeptide recycling). This profile resembles the transcriptional response of *L. plantarum* to p-coumaric acid (Reverón et al., 2012) and may similarly indicate that induction of the *opp* gene cluster is required for functions involved in the establishment of plant–microbe interactions, such as the sensing of plant peptide hormones and/or adhere to plant tissues, as previously reported for other microbial *opp* genes (Espinosa-Urgel et al., 2000; Monnet, 2003). The repression of a gene cluster encoding the ABC acetoin transporter was observed. Interestingly, this transporter is also downregulated in *L. plantarum* in presence of resveratrol (Reverón et al., 2018). Since acetoin can act as a signaling molecule for plant growth and development and trigger the induced systemic resistance (ISR) (Ryu et al., 2003), the repression of acetoin utilization in certain bacteria triggered by plant molecules such as OLE could be of utility to maintain a symbiotic association between the plant and bacterial members of the endophyte microbiome.

## Conclusion

**FIGURE 4:**
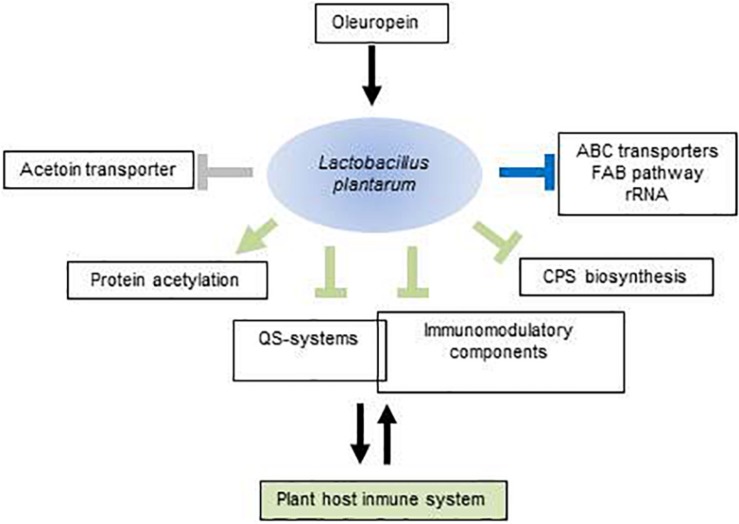
Schematic view of molecular components potentially involved in the *Lactobacillus plantarum*–plant host interaction that are modulated by oleuropein. Arrows represent upregulated components; perpendicular bars, downregulated components. In green, functions with potential to exert selective pressures on the plant immune system; in blue, functions mediating adaptation to oleuropein; in gray, functions affecting the effectiveness of plant developmental signals. Intersection of rectangles indicates that some QS-components act as immunomodulators. For more details, see text. FAB, fatty acid biosynthesis; QS, quorum sensing; CPS, polysaccharide.

This study increases the insights into the molecular mechanisms used by *L. plantarum* to respond to OLE (Figure 4). According to transcriptomic data this microorganism reduces growth, remodel membrane phospholipids and diminish the expression of several ABC transporters. Importantly, this study identifies OLE as a plant molecule with potential to act as a signal and impact on plant-bacterial combinations. Such managing role could be exerted by outfitting the expression of bacterial molecular players involved in this reciprocal interplay. Thus, OLE may help increase sensing of plant peptide hormones and adhesion to plant tissues by induction of *opp* genes. Notably, OLE may significantly modulate the plant immune responses to microbes such as *L. plantarum*, via down-regulation of bacterial immunomodulatory components, *cps* downregulation and increasing bacterial protein acetylation. By diminishing expression of the acetoin transporter and probably buffering ROS accumulation, *L. plantarum* could not undermine the effectiveness of acetoin and ROS as signaling molecules in plant development whereas the QS-antagonist activity of OLE probably help restrict microbial load in plant endosphere compartments. The controlled expression of all of these molecular players suggests that OLE acts a signaling molecule to accommodate and maintain a close association between beneficial microbes such as *L. plantarum* and the plant host. More broadly, this study indicates that some plant secondary metabolites, in this case OLE, could be recognized as signaling molecules by plant-associated bacteria and modify the expression of genes to interact with the plant host, thus extending the role of such molecules beyond the control of plant immunity. These understandings could pave the way to use OLE for a better management of the olive phytomicrobiome to obtain improved olive growth and resistance to stress.

## Data Availability Statement

The datasets generated for this study can be found in NCBI Gene Expression Omnibus, GSE90719.

## Author Contributions

RM, BR, and FF conceived and designed the research plans, and supervised the research. LS and IR performed most of the research. LP-V participated in RT-qPCR, and organized and submitted the data to repository databases. JO performed the functional analysis with DAVID. All authors analyzed the data. FF wrote the manuscript with contributions of all the authors.

## Conflict of Interest

The authors declare that the research was conducted in the absence of any commercial or financial relationships that could be construed as a potential conflict of interest.
