# Supplementary material for: Oleuropein Transcriptionally Primes Lactobacillus plantarum to Interact With Plant Hosts
Source: Front Microbiol. 2019 Sep 18;10:2177. doi: 10.3389/fmicb.2019.02177 (PMC6759512; doi:10.3389/fmicb.2019.02177)
Supplement: Supplementary file 1 [file Table_1.DOC]

**Additional file 1: Table S1.** Oligonucleotides used for qRT-PCR in this study.

| **Locus Tag^a^** | **Locus** | **Description** | **Primer sequence (5´ 3´)** |
| --- | --- | --- | --- |
| lp_3368 | *_* | multidrug transport protein, major facilitator superfamily (MFS), EmrB/QacA subfamily, N-terminal domain | F^b^: CGCCGTTGGTGCGATT  R^c^: CCGAATAATAACACGCCAAAGC |
| lp_0254 | *cysE* | serine O-acetyltransferase | F: TGGCGCCAACAGTAAAATTG  R: GGCGGGAACGCTGTCTAA |
| lp_2739 | *_* | ABC transporter ATP-binding protein | F: GGCGGGCAGCAAACAA  R: CCCTGGTTGCACATTGAAATT |
| lp_1424 | *_* | NADPH-dependent FMN reductase family protein | F: CACTGGTGATGCCAAATATTGAA  R: GCCCTGATCATCAAAAGCTTGT |
| lp_1425 | *_* | fumarate reductase/succinate dehydrogenase,FAD-binding flavoprotein; NADPH-dependent | F: CGGCAGCCCTGACCAA  R: GCCGGCATCAACGTAACG |
| lp_1426 | _ | hypothetical protein | F: TGACATCGACTGGCCCAAT  R: TGCCCTTTGTCAATGCTTCA |
| lp_1730 | *mapA* | maltose phosphorylase | F: GGGATACGCTTCCCGGTATT  R: AACCCGGGTCTTGTCTGGAT |
| lp_2960 | _ | lipase/esterase, subfamily of SGNH-hydrolases | F: CGCGTTCCAGGCAAAGA  R: GGCCGCGGCAATCAA |
| lp_2741 | _ | membrane protein | F: GGGCACCCTCCTCCTACTCT  R: TTGTCGTTCGGTACGATCGA |
| **Housekeeping and other internal control genes** | | |  |
| lp_2057 | *ldhD* | D-lactate dehydrogenase | F: AACCGCGACAATGTTTTGATT  R: TTGTGAACGGCAGTTTCAGTGT |
| lp_1021 | *rpoB* | DNA-directed RNA polymerase subunit beta | F: GGGTGTGCCTTCTCGTATGAA  R: CAGCCATCCCCAAATGCA |
| lp_1963 | *dnaG* | DNA primase DnaG | F: TCCGGAAGCAGTCGTCAAG  R: TCGCCGGCAAGTCAATGT |
| lp_0007 | *gyrA* | DNA gyrase, A subunit | F: CCCGACAGCAACGTCTTCA  R: GGCAGCTGGCGTTTGTTT |
| lp_2301 | *recA* | recombinase A | F: CGGCGGGCAGAACAGAT  R: TTTCCAAGCCACTCTTTTTTCG |
| lp_1962 | *rpoD* | RNA polymerase sigma factor RpoD | F: CGGATCCGCCAAATCG  R: CGTGATGGGTGGCGTAACTT |
| lp_0789 | *gapB* | glyceraldehyde 3-phosphate dehydrogenase | F: CTGGTGCTGCTAAGGCTCTTG  R: TGTGCATGGCCTTGTAATTTACC |
| lp_rRNA01 | 16srRNA | 16S ribosomal rRNA | F: GGGTAATCGGCCACATTGG  R: CTGCTGCCTCCCGTAGGA |

^a^ Designated gene number for the annotated *L. plantarum* WCFS1 genome

^b^ Forward

^c^ Reverse
