# Supplementary material for: Oleuropein Transcriptionally Primes Lactobacillus plantarum to Interact With Plant Hosts
Source: Front Microbiol. 2019 Sep 18;10:2177. doi: 10.3389/fmicb.2019.02177 (PMC6759512; doi:10.3389/fmicb.2019.02177)
Supplement: Supplementary file 2 [file Table_2.DOC]

**Additional file 2: Table S2.** Differentially expressed genes in *Lactobacillus plantarum* WCFS1 in presence of 15mM oleuropein

| **Gen ID** | **Locus** | **Fold Change ^a,b^** | **Description** | **COGs** | **Subcellular Localization** | **Pathway Prediction^c^** |
| --- | --- | --- | --- | --- | --- | --- |
| *lp_0009* | *rpsF* | -1,92 | 30S ribosomal protein S6 | Translation | Intracellular | No pathway |
| *lp_0010* |  | -2,82 | single-strand binding protein | Replication and repair | Intracellular | No pathway |
| *lp_0012* |  | -1,64 | phosphoesterase, DHH family | Translation, ribosomal structure and biogenesis | Multi-transmembrane | Sec-(SPI) |
| *lp_0013* | *rplI* | 1,98 | 50S ribosomal protein L9 | Translation, ribosomal structure and biogenesis | Intracellular | No pathway |
| *lp_0043* | *htrA* | 2,10 | serine protease HtrA | Post-translational modification, protein turnover, chaperone functions | N-terminally anchored (No CS) | Sec-(SPI) |
| *lp_0048* |  | -1,74 | uracil-DNA glycosylase family protein | Replication, recombination and repair | Intracellular | No pathway |
| *lp_0066* | *pgmB2* | 1,75 | beta-phosphoglucomutase | Multifunctional: Carbohydrate transport and metabolism / General function prediction only | Intracellular | No pathway |
| *lp_0091* |  | -1,60 | pyridoxamine 5\'-phosphate oxidase family protein, FMN-binding | Coenzyme transport and metabolism | Intracellular | No pathway |
| *lp_0114* | *thiD* | 1,61 | phosphomethylpyrimidine kinase & hydroxymethylpyrimidine kinase | Coenzyme transport and metabolism | Intracellular | No pathway |
| *lp_0151* |  | 1,77 | hypothetical protein | - | Intracellular | No pathway |
| *lp_0158* |  | 1,64 | hypothetical protein | - | Intracellular | No pathway |
| *lp_0168* | *dak1B* | -1,58 | dihydroxyacetone phosphotransferase,dihydroxyacetone binding subunit, dihydroxyacetone kinase subunit DhaK | Carbohydrate transport and metabolism | Intracellular | No pathway |
| *lp_0169* | *dak2* | -2,02 | dihydroxyacetone phosphotransferase,ADP-binding subunit | Carbohydrate metabolism and transport | Intracellular | No pathway |
| *lp_0171* | *gplF2* | 1,69 | dihydroxyacetone transport protein | Carbohydrate transport and metabolism | Multi-transmembrane | Sec-(SPI) |
| *lp_0197* |  | -1,72 | cell surface protein precursor, LPXTG-motif cell wall anchor | Cell wall/membrane/envelope biogenesis | LPxTG Cell-wall anchored | Sec-(SPI) |
| *lp_0202* |  | 1,69 | N-acetylglutamate synthase or related acetyltransferase, GNAT family | Amino acid transport and metabolism | Intracellular | No pathway |
| *lp_0204* | *serC* | 1,64 | phosphoserine aminotransferase | Multifunctional: Coenzyme transport and metabolism  Amino acid transport and metabolism | Intracellular | No pathway |
| *lp_0210* | *ack1* | -2,22 | acetate kinase | Energy production and conversion | Intracellular | No pathway |
| *lp_0230* | *pts2CB* | 2,36 | PTS system, mannitol-specific EIICB component | Carbohydrate transport and metabolism | Intracellular | No pathway |
| *lp_0247* | *pts3C* | -3,05 | PTS system, cellobiose-specific EIIC component | Carbohydrate transport and metabolism | Membrane | Sec-(SPI) |
| *lp_0254* | *cysE* | 4,57 | serine O-acetyltransferase | Amino acid transport and metabolism | Intracellular | No pathway |
| *lp_0255* | *metC1* | 3,94 | cystathionine beta-lyase / cystathionine gamma-lyase | Amino acid transport and metabolism | Intracellular | No pathway |
| *lp_0256* | *cysK* | 3,57 | cystathionine beta-synthase | Amino acid transport and metabolism | Intracellular | No pathway |
| *lp_0263* | *treA* | -1,61 | trehalose-6-phosphate hydrolase | Carbohydrate transport and metabolism | Intracellular | No pathway |
| *lp_0265* | *pts5ABC* | 1,73 | PTS system trehalose-specific transporter subunit IIBC | Carbohydrate transport and metabolism | Multi-transmembrane | Sec-(SPI) |
| *lp_0274* |  | -1,81 | TetR family transcriptional regulator | Transcription | Intracellular | No pathway |
| *lp_0281* |  | -1,61 | MerR family transcriptional regulator | Transcription | Intracellular | No pathway |
| *lp_0286* | *pts6C* | 2,10 | PTS system, cellobiose-specific EIIC component | Carbohydrate transport and metabolism | Multi-transmembrane | Sec-(SPI) |
| *lp_0298* | *-* | 2,10 | ABC transporter permease | Replication, recombination and repair | Multi-transmembrane | Sec-(SPI) |
| *lp_0299* | - | 1,47 | ABC transporter ATP-binding protein | Defense mechanisms | Intracellular | No pathway |
| *lp_0302* |  | -1,86 | extracellular transglycosylase |  | N-terminally anchored (with CS) | Sec-(SPI) |
| *lp_0316* | *potC* | -1,73 | spermidine/putrescine ABC transporter permease |  | Multi-transmembrane | Sec-(SPI) |
| *lp_0319* | *-* | -1,58 | spermidine/putrescine transport operon transcriptional regulator | Transcription | Intracellular | No pathway |
| *lp_0325* | - | -2,04 | acetoin transport repressor, GntR family | Transcription | Intracellular | No pathway |
| *lp_0326* | *-* | -2,06 | acetoin ABC transporter, ATP-binding protein | Defense mechanisms | Intracellular | No pathway |
| *lp_0327* | - | -1,79 | acetoin ABC transporter, permease protein |  | Multi-transmembrane | Sec-(SPI) |
| *lp_0329* | acdH | -1,59 | acetaldehyde dehydrogenase | Energy production and conversion | Intracellular | No pathway |
| *lp_0347* | - | -1,93 | PadR family transcriptional regulator | Transcription | Intracellular | No pathway |
| *lp_0348* | *-* | -1,93 | multidrug-efflux transporter | General function prediction only | Multi-transmembrane | Sec-(SPI) |
| *lp_0362* | *accB3* | 1,54 | acetyl-CoA carboxylase, biotin carboxyl carrier protein | Lipid transport and metabolism | Intracellular | No pathway |
| *lp_0370* | *glpK* | 1,87 | glycerol kinase | Energy production and conversion | Intracellular | No pathway |
| *lp_0371* | *glpD* | 2,01 | glycerol-3-phosphate dehydrogenase,FAD-dependent | Energy production and conversion | Intracellular | No pathway |
| *lp_0376* |  | -1,59 | membrane protein |  | Multi-transmembrane | Sec-(SPI) |
| *lp_0381* | *-* | -2,13 | ankyrin repeat family protein | General function prediction only | Intracellular | No pathway |
| *lp_0382* | - | -1,67 | membrane protein |  | Multi-transmembrane | Sec-(SPI) |
| *lp_0388* |  | -1,69 | hypothetical protein |  | Intracellular | No pathway |
| *lp_0404* | *plnL* | -1,93 | bacteriocin immunity protein PlnL |  | Multi-transmembrane | Sec-(SPI) |
| *lp_0405* | *plnK* | -1,78 | bacteriocin precursor peptide PlnK |  | Secreted via minor pathways (bacteriocin) (no CS) | Non-classical |
| *lp_0406* | *plnJ* | -2,30 | bacteriocin precursor peptide PlnJ |  | Secreted via minor pathways (bacteriocin) (no CS) | Non-classical |
| *lp_0412* | *plnP* | -1,82 | bacteriocin immunity protein PlnP,membrane-bound protease CAAX family |  | Multi-transmembrane | Sec-(SPI) |
| *lp_0415* | *plnA* | -4,45 | plantaricin A precursor peptide, induction factor |  | Secreted via minor pathways (bacteriocin) (no CS) | Non-classical |
| *lp_0416* | *plnB* | -6,13 | two-componet system histidine protein kinase PlnB; sensor protein | Signal Transduction | Multi-transmembrane | Sec-(SPI) |
| *lp_0417* | *plnC* | -4,23 | two-component response regulator PlnC,activator | Signal Transduction | Intracellular | No pathway |
| *lp_0418* | *plnD* | -2,85 | two-component response regulator PlnD,repressor | Transcription, Signal Transduction | Intracellular | No pathway |
| *lp_0419* | *plnI* | -1,64 | bacteriocin immunity protein PlnI,membrane-bound protease CAAX family |  | Multi-transmembrane | Sec-(SPI) |
| *lp_0421* | *plnF* | -1,53 | bacteriocin precursor peptide |  | Secreted via minor pathways (bacteriocin) (no CS) | Non-classical |
| *lp_0422* | *plnE* | -1,91 | bacteriocin precursor peptide PlnE |  | Secreted via minor pathways (bacteriocin) (no CS) | Non-classical |
| *lp_0423* | *plnG* | -1,60 | bacteriocin ABC-transporter, ATP-binding and permease protein PlnG | Defense mechanisms | Multi-transmembrane | Sec-(SPI) |
| *lp_0424* | *plnH* | 1,63 | bacteriocin ABC transporter, accessory factor PlnH |  | N-terminally anchored (No CS) | Sec-(SPI) |
| *lp_0428* | *plnV* | -1,62 | membrane protein plnV |  | Multi-transmembrane | Sec-(SPI) |
| *lp_0447* | *mvaA* | 1,69 | hydroxymethylglutaryl-CoA reductase | Lipid transport and metabolism | Intracellular | No pathway |
| *lp_0449* |  | 1,64 | NAD-dependent deacetylase, SIR2 family | Posttranslational modification, protein turnover, chaperones | Intracellular | No pathway |
| *lp_0463* |  | 1,56 | zinc/iron ABC transporter, ATP-binding protein | Inorganic ion transport and metabolism | Intracellular | No pathway |
| *lp_0490* | *-* | -2,04 | NADPH-dependent FMN reductase | General function prediction only | Intracellular | No pathway |
| *lp_0513* |  | 1,67 | hypothetical protein |  | Intracellular | No pathway |
| *lp_0524* |  | -1,79 | toxin-antitoxin system, toxin component DNA-binding protein, PemK/MazF family | Signal transduction mechanisms | Intracellular | No pathway |
| *lp_0537* | ldhL1 | 1,62 | L-lactate dehydrogenase | Energy production and conversion | Intracellular | No pathway |
| *lp_0559* |  | -1,80 | ribosyl nicotinamide transporter, PnuC-like | Coenzyme transport and metabolism | Multi-transmembrane | Sec-(SPI) |
| *lp_0565* | nadC1 | -1,90 | nicotinate phosphoribosyltransferase | Coenzyme transport and metabolism | Intracellular | No pathway |
| *lp_0568* |  | 1,66 | hypothetical protein |  | Intracellular | No pathway |
| *lp_0587* | pts10B | 1,75 | PTS system, mannose-specific EIIB component | Carbohydrate transport and metabolism | Intracellular | No pathway |
| *lp_0589* | accB1 | 1,87 | acetyl-CoA carboxylase, biotin carboxyl carrier protein | Lipid transport and metabolism | Intracellular | No pathway |
| *lp_0591* | accD1 | 1,88 | acetyl-CoA carboxylase, carboxyl transferase subunit beta | Lipid transport and metabolism | Intracellular | No pathway |
| *lp_0592* | accA1 | 1,75 | acetyl-CoA carboxylase, carboxyl transferase subunit alpha | Lipid transport and metabolism | Intracellular | No pathway |
| *lp_0616* | secE | -1,79 | preprotein translocase subunit SecE | Intracellular trafficking, secretion, and vesicular transport | N-terminally anchored (No CS) | Sec-(SPI) |
| *lp_0624* | 1 | -1,59 | prophage P1 protein 1, integrase | Replication, recombination and repair | Intracellular | No pathway |
| *lp_0625* |  | -1,63 | prophage P1 protein 2, mitogenic factor, cell surface lipoprotein | - | Lipid anchored | Sec-(SPII) |
| *lp_0630* | *-* | -2,02 | prophage P1 protein 7 | Mobilome: prophages, transposons | Intracellular | No pathway |
| *lp_0631* | *-* | -1,95 | prophage P1 protein 8, phage Cro/CI family transcriptional regulator | Transcription | Intracellular | No pathway |
| *lp_0667* |  | -1,58 | prophage P1 protein 44 | Mobilome: prophages, transposons | Intracellular | No pathway |
| *lp_0674* |  | -1,72 | prophage P1 protein 51 | Mobilome: prophages, transposons | Intracellular | No pathway |
| *lp_0686* |  | -1,67 | prophage P1 protein 63 | Mobilome: prophages, transposons | Intracellular | No pathway |
| *lp_0690* |  | -1,62 | membrane protein | Function unknown | Multi-transmembrane | Sec-(SPI) |
| *lp_0691* |  | 1,60 | hypothetical protein | General function prediction only | Intracellular | No pathway |
| *lp_0692* | *nrdF* | 1,77 | ribonucleoside-diphosphate reductase, beta chain | Nucleotide transport and metabolism | Intracellular | No pathway |
| *lp_0693* | *nrdE* | 1,72 | ribonucleoside-diphosphate reductase subunit alpha | Nucleotide transport and metabolism | Intracellular | No pathway |
| *lp_0720* | *rimI2* | -1,74 | ribosomal-protein-alanine N-acetyltransferase | General function prediction only | Intracellular | No pathway |
| *lp_0727* | *groES* | 2,1 | co-chaperonin | Posttranslational modification, protein turnover, chaperones | Intracellular | No pathway |
| *lp_0728* | *groEL* | 1,62 | GroEL chaperonin | Post-translational modification, protein turnover, chaperone functions | Intracellular | No pathway |
| *lp_0752* |  | -1,83 | stress-responsive transcription regulator | Multifunctional:  Transcription /  Signal transduction mechanisms | Multi-transmembrane | Sec-(SPI) |
| *lp_0791* | *tpiA* | 1,61 | triosephosphate isomerase | Carbohydrate transport and metabolism | Intracellular | No pathway |
| *lp_0797* |  | -1,72 | exoribonuclease II | Transcription | Intracellular | No pathway |
| *lp_0827* |  | -1,69 | hypothetical protein | - | Intracellular | No pathway |
| *lp_0828* |  | -1,84 | hypothetical protein | - | Intracellular | No pathway |
| *lp_0842* | *ppk* | -1,55 | polyphosphate kinase | Inorganic ion transport and metabolism | Intracellular | No pathway |
| *lp_0843* | *ppx3* | -1,68 | exopolyphosphatase | Multifunctional: Nucleotide transport and metabolism Inorganic ion transport and metabolism | Intracellular | No pathway |
| *lp_0861* |  | 1,75 | branched-chain amino acid permease | Amino Acid metabolism and transport | Multi-transmembrane | Sec-(SPI) |
| *lp_0918* |  | -1,95 | superfamily I DNA/RNA helicase | Replication, recombination and repair | Intracellular | No pathway |
| *lp_0929* | *asp1* | 2,16 | alkaline shock protein | Function unknown | Intracellular | No pathway |
| *lp_0956* | *asnC* | 1,62 | asparagine-tRNA synthetase | Translation, ribosomal structure and biogenesis | Intracellular | No pathway |
| *lp_0966* |  | -1,72 | NADPH-dependent FMN reductase | General function prediction only | Multi-transmembrane | Sec-(SPI) |
| *lp_0997* | *murE1* | 1,61 | cold shock protein CspC | Transcription | Intracellular | No pathway |
| *lp_1003* |  | 1,61 | acetyltransferase GNAT family | Translation, ribosomal structure and biogenesis  Transcription | Intracellular | No pathway |
| *lp_1033* | *rplC* | -2,87 | 50S ribosomal protein L3 | Translation | Intracellular | No pathway |
| *lp_1034* | *rplD* | -1,6 | 50S ribosomal protein L4 | Translation | Intracellular | No pathway |
| *lp_1035* | *rplW* | -2,26 | 50S ribosomal protein L23 | Translation | Intracellular | No pathway |
| *lp_1036* | *rplB* | -3,68 | 50S ribosomal protein L2 | Translation | Intracellular | No pathway |
| *lp_1038* | *rpsS* | -1,9 | 30S ribosomal protein S19 | Translation | Intracellular | No pathway |
| *lp_1039* | *rplV* | -2,32 | 50S ribosomal protein L22 | Translation | Intracellular | No pathway |
| *lp_1045* | *rplN* | -2,22 | 50S ribosomal protein L14 | Translation | Intracellular | No pathway |
| *lp_1053* | *rpsE* | -2 | 30S ribosomal protein S5 | Translation | Intracellular | No pathway |
| *lp_1069* | *ndh2* | 1,71 | NADH dehydrogenase, membrane-anchored | Energy production and conversion | Multi-transmembrane | Sec-(SPI) |
| *lp_1106* | *citC* | 1,66 | [citrate (pro-3S)-lyase] ligase | Energy production and conversion | Intracellular | No pathway |
| *lp_1107* | *citD* | 1,60 | citrate lyase, gamma chain, acyl carrier protein | Energy production and conversion | Intracellular | No pathway |
| *lp_1108* | *citE* | 1,57 | citrate lyase, beta chain | Carbohydrate transport and metabolism | Intracellular | No pathway |
| *lp_1118* | *mleS* | 1,73 | malolactic protein | Energy production and conversion | Multi-transmembrane | Sec-(SPI) |
| *lp_1120* |  | 1,73 | amino acid transport protein | Amino acid transport and metabolism | Multi-transmembrane | Sec-(SPI) |
| *lp_1180* | *cps1D* | -1,80 | glycosyltransferase | - | Intracellular | No pathway |
| *lp_1184* | *cps1H* | -1,81 | glycosyltransferase (rhamnosyltransferase),family 2 (GT2) | Cell wall/membrane/envelope biogenesis | Intracellular | No pathway |
| *lp_1185* | *cps1I* | -2,08 | polysaccharide polymerase | - | Intracellular | No pathway |
| *lp_1186* | *rfbA* | -1,63 | glucose-1-phosphate thymidylyltransferase | Cell wall/membrane/envelop biogenesis | Intracellular | No pathway |
| *lp_1197* | *cps2A* | -1,68 | polysaccharide biosynthesis protein, chain length regulator | Cell wall/membrane/envelope biogenesis | Multi-transmembrane | Sec-(SPI) |
| *lp_1198* | *cps2B* | -1,84 | polysaccharide biosynthesis protein, regulator | Cell cycle control, cell division, chromosome partitioning | Intracellular | No pathway |
| *lp_1201* | *cps2E* | -1,77 | priming glycosyltransferase, polyprenyl glycosylphosphotransferase | Cell wall/membrane/envelope biogenesis | N-terminally anchored (No CS) | Sec-(SPI) |
| *lp_1202* | *cps2F* | -1,69 | glycosyltransferase, family 1 (GT1) | Cell wall/membrane/envelope biogenesis | Intracellular | No pathway |
| *lp_1203* | *cps2G* | -1,68 | polysaccharide biosynthesis protein | - | Intracellular | No pathway |
| *lp_1207* | *cps2K* | -2,27 | polysaccharide biosynthesis protein | Replication, recombination and repair | Intracellular | No pathway |
| *lp_1213* |  | -1,80 | hypothetical protein | - | Intracellular | No pathway |
| *lp_1215* | *cps3A* | -1,79 | glycosyltransferase | General function prediction only | Intracellular | No pathway |
| *lp_1219* | *glf2* | -1,74 | UDP-galactopyranose mutase | Cell wall/membrane/envelope biogenesis | N-terminally anchored (No CS) | Sec-(SPI) |
| *lp_1222* | *cps3F* | -1,91 | polysaccharide polymerase | - | Multi-transmembrane | Sec-(SPI) |
| *lp_1224* | *cps3G* | -2,26 | polysaccharide biosynthesis membrane protein | - | Intracellular | No pathway |
| *lp_1233* |  | -1,69 | priming glycosyltransferase,undecaprenyl-phosphate beta-glucosephosphotransferase | Cell wall/membrane/envelope biogenesis | N-terminally anchored (No CS) | Sec-(SPI) |
| *lp_1253* | *gshR2* | 1,67 | glutathione reductase | Energy production and conversion | Intracellular | No pathway |
| *lp_1261* | *oppA* | 2,46 | oligopeptide ABC transporter substrate binding protein | Amino acid transport and metabolism | Lipid anchored | Sec-(SPII) |
| *lp_1262* | *oppB* | 2,32 | oligopeptide ABC transporter permease | Inorganic ion transport and metabolism,Amino Acid metabolis and transport | Multi-transmembrane | Sec-(SPI) |
| *lp_1263* | *oppC* | 2,30 | oligopeptide ABC transporter permease | Amino acid transport and metabolism & Inorganic ion transport and metabolism | Multi-transmembrane | Sec-(SPI) |
| *lp_1264* | *oppD* | 2,21 | oligopeptide ABC transporter ATP-binding protein | Amino acid transport and metabolism & Inorganic ion transport and metabolism | Intracellular | No pathway |
| *lp_1265* | *oppF* | 1,76 | oligopeptide ABC transporter ATP-binding protein | General functional prediction only | Intracellular | No pathway |
| *lp_1268* |  | -1,90 | integrase/recombinase | Replication, recombination and repair | Intracellular | No pathway |
| *lp_1269* | *clpE* | 2,17 | ATP-dependent Clp protease, ATP-binding, subunit ClpE | Posttranslational modification, protein turnover, chaperones | Intracellular | No pathway |
| *lp_1273* | *hpr* | 1,65 | phosphocarrier protein Hpr | Carbohydrate transport and metabolism | Intracellular | No pathway |
| *lp_1310* |  | -1,64 | hypothetical protein | - | Intracellular | No pathway |
| *lp_1311* | *tagE2* | -1,73 | poly(glycerol-phosphate) alpha-glucosyltransferase | Cell wall/membrane/envelope biogenesis | Intracellular | No pathway |
| *lp_1357* |  | 1,68 | extracellular protein, membrane-anchored | - | N-terminally anchored (No CS) | Sec-(SPI) |
| *lp_1377* |  | 1,84 | hypothetical protein | - | Intracellular | No pathway |
| *lp_1388* | *-* | -2,30 | hypothetical protein | - | Intracellular | No pathway |
| *lp_1395* |  | -1,70 | hypothetical protein | - | Intracellular | No pathway |
| *lp_1398* | *pts15A* | -1,85 | PTS system, beta-glucosides-specific EIIA component | Carbohydrate transport and metabolism | Intracellular | No pathway |
| *lp_1415* |  | -1,70 | hypothetical protein | - | Intracellular | No pathway |
| *lp_1417* |  | -1,68 | membrane protein | - | Intracellular | No pathway |
| *lp_1424* | *-* | 6,08 | NADPH-dependent FMN reductase family protein | General function prediction only | Intracellular | No pathway |
| *lp_1425* | *-* | 5,81 | fumarate reductase/succinate dehydrogenase,FAD-binding flavoprotein; NADPH-dependent | General function prediction only | Intracellular | No pathway |
| *lp_1426* | *-* | 2,63 | hypothetical protein | Cell wall/membrane/envelope biogenesis | Intracellular | No pathway |
| *lp_1505* | *-* | -1,85 | membrane protein | - | Multi-transmembrane | Sec-(SPI) |
| *lp_1506* | - | -1,91 | Cro/CI family transcriptional regulator | Transcription | Intracellular | No pathway |
| *lp_1512* | *dnaB* | 1,72 | replication initiation and membrane attachment protein DnaB | Replication, recombination and repair | Intracellular | No pathway |
| *lp_1521* |  | -1,83 | oxidoreductase | Multifunctional:  Energy production and conversión  General function prediction only | Intracellular | No pathway |
| *lp_1535* |  | -1,67 | hypothetical protein | - | Intracellular | No pathway |
| *lp_1568* | *pbp2B1* | -1,58 | transpeptidase (penicillin binding protein 2B) | Cell cycle control, cell division, chromosome partitioning  Cell wall, membrane and envelope biogenesis | N-terminally anchored (No CS) | Sec-(SPI) |
| *lp_1572* |  | -1,72 | hypothetical protein | - | Intracellular | No pathway |
| *lp_1573* | *glk* | -1,59 | transcription regulator; sugar kinase, ROK family | Multifunctional:  Transcription, Carbohydrate transport and metabolism | Intracellular | No pathway |
| *lp_1579* | *miaA* | 1,71 | tRNA isopentenylpyrophosphate transferase | Translation, ribosomal structure and biogenesis | N-terminally anchored (No CS) | Sec-(SPI) |
| *lp_1594* | *rpmA* | 1,81 | 50S ribosomal protein L27 | Translation, ribosomal structure and biogenesis | Intracellular | No pathway |
| *lp_1625* |  | -1,75 | hypothetical protein | - | Intracellular | No pathway |
| *lp_1626* |  | -1,65 | hypothetical protein | - | Intracellular | No pathway |
| *lp_1669* | *-* | -1,83 | AraC family transcriptional regulator | Transcription | Intracellular | No pathway |
| *lp_1670* | *fabZ1* | -2,47 | (3R)-hydroxyacyl-[acyl carrier protein] dehydratase | Lipid transport and metabolism | Intracellular | No pathway |
| *lp_1671* | *fabH2* | -3,24 | 3-oxoacyl-ACP synthase | Lipid transport and metabolism | Intracellular | No pathway |
| *lp_1672* | *acpA2* | -5,70 | acyl carrier protein | Lipid transport and metabolism & Secondary metabolites biosynthesis, transport and catabolism | Intracellular | No pathway |
| *lp_1673* | *fabD* | -3,36 | [acyl-carrier protein] S-malonyltransferase | Lipid transport and metabolism | Intracellular | No pathway |
| *lp_1674* | *fabG1* | -4,35 | 3-oxoacyl-ACP reductase | Multifunctional: Lipid metabolism, Secondary structure, General functional prediction only | Intracellular | No pathway |
| *lp_1675* | *fabF* | -2,04 | 3-oxoacyl-ACP synthase | Lipid transport and metabolism & Secondary metabolites biosynthesis, transport and catabolism | Intracellular | No pathway |
| *lp_1676* | *accB2* | -3,32 | acetyl-CoA carboxylase, biotin carboxyl carrier protein | Lipid transport and metabolism | Intracellular | No pathway |
| *lp_1677* | *fabZ2* | -2,5 | (3R)-hydroxymyristoyl-ACP dehydratase | Lipid transport and metabolism | Intracellular | No pathway |
| *lp_1678* | *accC2* | -3,84 | acetyl-CoA carboxylase, biotin carboxylase subunit | Lipid transport and metabolism | Intracellular | No pathway |
| *lp_1679* | *accD2* | -2,40 | acetyl-CoA carboxylase, carboxyl transferase subunit beta | Lipid transport and metabolism | Intracellular | No pathway |
| *lp_1681* | *fabI* | -2,29 | enoyl-[acyl-carrier protein] reductase (NADH) | Lipid transport and metabolism | Intracellular | No pathway |
| *lp_1682* | *-* | -1,94 | phosphopantetheinyltransferase | Coenzyme transport and metabolism | Intracellular | No pathway |
| *lp_1685* | *-* | -2,00 | LysR family transcriptional regulator | Transcription | Intracellular | No pathway |
| *lp_1686* | *-* | -1,92 | acyl-CoA hydrolase | Lipid transport and metabolism | Intracellular | No pathway |
| *lp_1694* |  | -1,67 | hypothetical protein | - | Intracellular | No pathway |
| *lp_1695* |  | -1,85 | membrane protein | - | Multi-transmembrane | Sec-(SPI) |
| *lp_1696* | *cfa1* | -1,72 | cyclopropane-fatty-acyl-phospholipid synthase | Cell wall, membrane and envelope biogenesis | Intracellular | No pathway |
| *lp_1699* |  | -1,84 | hypothetical protein | - | Intracellular | No pathway |
| *lp_1700* | *tspO* | 1,63 | sensory protein | Signal transduction mechanisms | Multi-transmembrane | Sec-(SPI) |
| *lp_1702* |  | 1,81 | membrane protein | Function unknown | Multi-transmembrane | Sec-(SPI) |
| *lp_1703* |  | 1,56 | hypothetical protein | Lipid transport and metabolism &  General function prediction only | Intracellular | No pathway |
| *lp_1704* |  | 1,61 | membrane protein | - | Multi-transmembrane | Sec-(SPI) |
| *lp_1705* |  | 1,55 | hypothetical protein | - | N-terminally anchored (No CS) | Sec-(SPI) |
| *lp_1706* |  | 1,70 | membrane protein | - | Multi-transmembrane | Sec-(SPI) |
| *lp_1708* |  | 1,67 | hypothetical protein | - | Intracellular | No pathway |
| *lp_1729* | *malT* | 3,35 | carbohydrate/proton transporter | General function prediction only | Multi-transmembrane | Sec-(SPI) |
| *lp_1730* | *mapA* | 2,58 | maltose phosphorylase | Carbohydrate transport and metabolism | Intracellular | No pathway |
| *lp_1731* | *galM2* | 2,48 | aldose 1-epimerase | Carbohydrate transport and metabolism | Intracellular | No pathway |
| *lp_1736* | *-* | 1,89 | hypothetical protein | - | Intracellular | No pathway |
| *lp_1747* |  | 1,67 | hypothetical protein | Signal transduction mechanisms | Intracellular | No pathway |
| *lp_1773* |  | 1,62 | membrane protein | - | Multi-transmembrane | Sec-(SPI) |
| *lp_1776* |  | -1,61 | oxidoreductase | Amino acid transport and metabolism | Intracellular | No pathway |
| *lp_1806* |  | 1,58 | hypothetical protein | - | Intracellular | No pathway |
| *lp_1811* |  | 1,65 | amino acid transport protein | Amino acid transport and metabolism | Multi-transmembrane | Sec-(SPI) |
| *lp_1816* | *ispD* | 2,89 | 3 2-C-methyl-D-erythritol 4-phosphate cytidylyltransferase | Lipid transport and metabolism | Intracellular | No pathway |
| *lp_1817* | *-* | 2,84 | tarJ ribitol-5-phosphate 2-dehydrogenase | Amino acid transport and metabolism | Intracellular | No pathway |
| *lp_1818* | *tagB1* | 1,53 | teichoic acid biosynthesis protein | Cell wall, membrane and envelope biogenesis | Intracellular | No pathway |
| *lp_1853* | *rnhB* | 1,73 | ribonuclease HII | Replication, recombination and repair | Intracellular | No pathway |
| *lp_1903* | *clpB* | 1,74 | ATP-dependent Clp protease, ATP-binding subunit ClpB | Posttranslational modification, protein turnover, chaperones | Intracellular | No pathway |
| *lp_1904* | *pepT* | 1,61 | peptidase T | Amino acid transport and metabolism | Intracellular | No pathway |
| *lp_1905* | *-* | 1,70 | hypothetical protein | Function unknown | Intracellular | No pathway |
| *lp_1914* | *-* | -1,84 | transcription regulator | Transcription | Intracellular | No pathway |
| *lp_1921* | *-* | 1,83 | transport protein | Carbohydrate transport and metabolism | Multi-transmembrane | Sec-(SPI) |
| *lp_1922* | *-* | 1,79 | transcription regulator | Transcription | Intracellular | No pathway |
| *lp_1923* | *dapE1* | 1,59 | succinyl-diaminopimelate desuccinylase | Amino acid transport and metabolism | Intracellular | No pathway |
| *lp_1928* | *nrpR2* | 1,58 | negative regulator of proteolysis | Inorganic ion transport and metabolism | Intracellular | No pathway |
| *lp_1959* | *-* | -1,69 | transcription regulator | Transcription | Intracellular | No pathway |
| *lp_1962* | *rpoD* | 1,71 | RNA polymerase sigma factor RpoD | Transcription | Intracellular | No pathway |
| *lp_1979* | *msrA4* | 1,71 | protein-methionine-S-oxide reductase | Posttranslational modification, protein turnover, chaperones | Intracellular | No pathway |
| *lp_1980* | *aspS* | 1,70 | aspartyl-tRNA synthetase | Translation, ribosomal structure and biogenesis | Intracellular | No pathway |
| *lp_2026* | *dnaJ* | 1,62 | molecular chaperone DnaJ | Posttranslational modification, protein turnover, chaperones | Intracellular | No pathway |
| *lp_2027* | *dnaK* | 1,97 | molecular chaperone DnaK | Posttranslational modification, protein turnover, chaperones | Intracellular | No pathway |
| *lp_2028* | *grpE* | 1,93 | heat shock protein GrpE | Posttranslational modification, protein turnover, chaperones | Intracellular | No pathway |
| *lp_2029* | *hrcA* | 2,57 | heat-inducible transcription repressor | Transcription | Intracellular | No pathway |
| *lp_2034* | *tyrA* | 1,59 | prephenate dehydrogenase | Amino acid transport and metabolism | Intracellular | No pathway |
| *lp_2035* | *aroE* | 1,62 | 3-phosphoshikimate 1-carboxyvinyltransferase | Amino acid transport and metabolism | Intracellular | No pathway |
| *lp_2036* | *-* | 1,81 | hypothetical protein |  | Intracellular | No pathway |
| *lp_2037* | *aroF* | 2,09 | chorismate synthase | Amino acid transport and metabolism | Intracellular | No pathway |
| *lp_2038* | *-* | 1,63 | transport protein | Amino acid transport and metabolism | Multi-transmembrane | Sec-(SPI) |
| *lp_2057* | *ldhD* | -1,65 | D-lactate dehydrogenase la otra sonda positivo cerca de 1,15 | Multifunctional:  Energy production and conversion &  Coenzyme transport and metabolism | Intracellular | No pathway |
| *lp_2063* | *lexA* | -2,25 | transcription repressor and protease LexA of the SOS regulon | Transcription & Signal transduction mechanisms | Intracellular | No pathway |
| *lp_2087* | *recJ* | 1,91 | single-strand DNA-specific exonuclease RecJ | Replication, recombination and repair | Intracellular | No pathway |
| *lp_2095* | *fruR* | 1,87 | transcription regulator of fructose operon | Transcription & Carbohydrate transport and metabolism | Intracellular | No pathway |
| *lp_2096* | *fruK* | 1,57 | 1-phosphofructokinase | Carbohydrate transport and metabolism | Intracellular | No pathway |
| *lp_2097* | *pts16ABC* | 1,88 | fructose PTS, EIIABC | Carbohydrate transport and metabolism | Multi-transmembrane | Sec-(SPI) |
| *lp_2099* | *cps4J* | -1,85 | polysaccharide repeat unit transporter (flippase) | General function prediction only | Multi-transmembrane | Sec-(SPI) |
| *lp_2100* | *cps4I* | -1,53 | glycosyltransferase, family 2 (GT2) | Cell wall/membrane/ envelope biogenesis | Intracellular | No pathway |
| *lp_2101* | *cps4H* | -1,70 | polysaccharide polymerase | Uncategorized | Multi-transmembrane | Sec-(SPI) |
| *lp_2102* | *cps4G* | -1,84 | glycosyltransferase, family 1 (GT1) | Cell wall/membrane/ envelope biogenesis | Intracellular | No pathway |
| *lp_2103* | *cps4F* | -1,93 | glycosyltransferase, family 1 (GT1) | Cell wall/membrane/ envelope biogenesis | Intracellular | No pathway |
| *lp_2104* | *cps4E* | -1,95 | polysaccharide biosynthesis polyprenyl glycosylphosphotransferase, priming glycosyltransferase | Cell wall/membrane/ envelope biogenesis | N-terminally anchored (No CS) | Sec-(SPI) |
| *lp_2105* | *galE3* | -2,92 | UDP N-acetyl glucosamine 4-epimerase, NAD dependent | Carbohydrate transport and metabolism & Cell wall/membrane/envelope biogenesis | Intracellular | No pathway |
| *lp_2106* | *cps4C* | -1,96 | exopolysaccharide biosynthesis protein | Multifunctional: Cell wall/membrane/envelope biogenesis & Carbohydrate metabolism and transport | Intracellular | No pathway |
| *lp_2107* | *cps4B* | -2,52 | polysaccharide biosynthesis protein | Cell cycle control, cell division, chromosome partitioning | Intracellular | No pathway |
| *lp_2108* | *cps4A* | -2,51 | polysaccharide biosynthesis protein, chain length regulator | Cell wall/membrane/ envelope biogenesis | Multi-transmembrane | Sec-(SPI) |
| *lp_2113* |  | 1,87 | hypothetical protein | Multifunctional: Carbohydrate metabolism and transport & Cell wall/membrane/ envelope biogenesis | Intracellular | No pathway |
| *lp_2122* |  | 1,70 | metallo-beta-lactamase superfamily protein | General function prediction only | Intracellular | No pathway |
| *lp_2151* | *pdhD* | 1,82 | dihydrolipoamide dehydrogenase | Energy production and conversion | Intracellular | No pathway |
| *lp_2153* | *pdhB* | 1,81 | pyruvate dehydrogenase complex, E1 component, beta subunit | Energy production and conversion | Intracellular | No pathway |
| *lp_2154* | *pdhA* | 1,55 | pyruvate dehydrogenase complex, E1 component, alpha subunit | Energy production and conversion | Intracellular | No pathway |
| *lp_2155* | *def* | 1,56 | peptide deformylase | Translation, ribosomal structure and biogenesis | Intracellular | No pathway |
| *lp_2156* | *-* | 1,61 | hypothetical protein | - | N-terminally anchored (No CS) | Sec-(SPI) |
| *lp_2157* | *-* | 1,75 | hypothetical protein | Function unknown | Intracellular | No pathway |
| *lp_2158* | *-* | 1,54 | metallo-beta-lactamase superfamily protein | General function prediction only | Intracellular | No pathway |
| *lp_2192* | *-* | 1,96 | cell division protein | Function unknown | Intracellular | No pathway |
| *lp_2193* | *ftsZ* | 1,73 | cell division protein FtsZ | Cell cycle control, cell division, chromosome partitioning | Intracellular | No pathway |
| *lp_2194* | *ftsA* | 1,68 | cell division protein FtsA | Cell cycle control, cell division, chromosome partitioning | Intracellular | No pathway |
| *lp_2203* | *mraZ* | -1,59 | cell division protein MraZ | Function unknown | Intracellular | No pathway |
| *lp_2216* | *rpsN2* | 1,63 | ribosomal protein S14-2 | Translation, ribosomal structure and biogenesis | Intracellular | No pathway |
| *lp_2228* | *nrpR3* | 1,89 | negative regulator of proteolysis | Inorganic ion transport and metabolism | Intracellular | No pathway |
| *lp_2231b* | *-* | -1,66 | polyribonucleotide nucleotidyltransferase | Translation, ribosomal structure and biogenesis | Intracellular | No pathway |
| *lp_2240* | *-* | -1,97 | amino acid transport protein | Amino acid transport and metabolism | Multi-transmembrane | Sec-(SPI) |
| *lp_2273* | *-* | 1,68 | hypothetical protein | Function unknown | Intracellular | No pathway |
| *lp_2276* | *-* | -1,82 | hypothetical protein | - | Intracellular | No pathway |
| *lp_2292* | *-* | -1,55 | hypothetical protein | - | Intracellular | No pathway |
| *lp_2305* | *-* | -1,71 | zinc-dependent proteinase | General function prediction only | Intracellular | No pathway |
| *lp_2308* | *thrA2* | 1,77 | aspartate kinase | Amino acid transport and metabolism | Intracellular | No pathway |
| *lp_2339* | *-* | -1,64 | hypothetical protein | - | Multi-transmembrane | Sec-(SPI) |
| *lp_2443* | *-* | -2,61 | prophage P2a protein 14 | Function unknown | Intracellular | No pathway |
| *lp_2449* | *hicD3* | -1,60 | prophage Lp2 protein 8 | Amino acid transport and metabolism | Intracellular | No pathway |
| *lp_2460* | *-* | -2,15 | prophage Lp3 protein 21 | - | Intracellular | No pathway |
| *lp_2469* | *atpE* | -1,76 | prophage Lp3 protein 12 | - | N-terminally anchored (No CS) | Sec-(SPI) |
| *lp_2531* | *pts18CBA* | -1,67 | N-acetylglucosamine and glucose PTS, EIICBA | Carbohydrate transport and metabolism | Multi-transmembrane | Sec-(SPI) |
| *lp_2544* | *npr2* | 1,72 | NADH peroxidase | General function prediction only | Intracellular | No pathway |
| *lp_2601* | *-* | -1,84 | HAD superfamily hydrolase | General function prediction only | Intracellular | No pathway |
| *lp_2614* | *-* | 1,90 | ABC transporter ATP-binding protein | Defense mechanisms | Intracellular | No pathway |
| *lp_2631* | *-* | 1,85 | lipase/esterase | Amino acid transport and metabolism | Intracellular | No pathway |
| *lp_2638* | *-* | 3,65 | membrane protein | General function prediction only | Multi-transmembrane | Sec-(SPI) |
| *lp_2647* | *pts19A* | -1,88 | N-acetylglucosamine/galactosamine PTS, EIIA | Carbohydrate transport and metabolism | Intracellular | No pathway |
| *lp_2650* | *pts19B* | -1,70 | PTS system N-acetylgalactosamine-specific transporter subunit IIB | Carbohydrate transport and metabolism | Intracellular | No pathway |
| *lp_2665* | *rrp8* | -1,99 | response regulator | Transcription and Signal Transduction | Intracellular | No pathway |
| *lp_2666* | *-* | 1,64 | hypothetical protein | - | Intracellular | No pathway |
| *lp_2708* | *pucR* | 1,90 | pucR purine transport regulator | Secondary metabolites biosynthesis, transport and catabolism & Signal transduction mechanisms | Intracellular | No pathway |
| *lp_2710* | *pucK* | 1,81 | xanthine / uracil transport protein | Nucleotide metabolism and transport | Multi-transmembrane | Sec-(SPI) |
| *lp_2728* | *purK1* | -1,64 | phosphoribosylaminoimidazole carboxylase ATPase subunit | Nucleotide transport and metabolism | Intracellular | No pathway |
| *lp_2738* | *-* | -2,16 | L-asparaginase | Amino acid transport and metabolism & Translation, ribosomal structure and biogenesis | Intracellular | No pathway |
| *lp_2739* | *-* | -20,40 | ABC transporter ATP-binding protein | Defense mechanisms | Intracellular | No pathway |
| *lp_2740* | *-* | -16,62 | ABC transporter permease | Defense mechanisms | Multi-transmembrane | Sec-(SPI) |
| *lp_2741* | *-* | -3,05 | membrane protein |  | Multi-transmembrane | Sec-(SPI) |
| *lp_2742* | *-* | -2,91 | GntR family transcriptional regulator | Transcription | Intracellular | No pathway |
| *lp_2743* | *-* | -2,41 | ABC transporter ATP-binding protein | Defense mechanisms | Intracellular | No pathway |
| *lp_2744* | *-* | -2,58 | ABC transporter permease |  | Multi-transmembrane | Sec-(SPI) |
| *lp_2773* | *-* | 1,75 | ABC transporter permease | Defense mechanisms | Multi-transmembrane | Sec-(SPI) |
| *lp_2774* | *-* | 1,68 | ABC transporter ATP-binding protein | Defense mechanisms | Intracellular | No pathway |
| *lp_2777* | *pbg4* | 2,10 | 6-phospho-beta-glucosidase | Carbohydrate transport and metabolism | Intracellular | No pathway |
| *lp_2778* | *pbg5* | 1,79 | 6-phospho-beta-glucosidase | Carbohydrate transport and metabolism | Intracellular | No pathway |
| *lp_2780* | *pts20A* | 2,17 | PTS system, cellobiose-specific EIIA component | Carbohydrate transport and metabolism | Intracellular | No pathway |
| *lp_2781* | *-* | 2,16 | pts20B PTS system, cellobiose-specific EIIB component | Carbohydrate transport and metabolism | N-terminally anchored (with CS) | Sec-(SPI) |
| *lp_2810* | *-* | -2,1 | glycosyl hydrolase family protein / lisin | Cell wall/membrane/ envelope biogenesis | N-terminally anchored (No CS) | Sec-(SPI) |
| *lp_2857* |  | 1,71 | ABC transporter, ATP-binding protein,C-terminal domain | Multifunctional:  Nucleotide transport and metabolism & Inorganic ion transport and metabolism |  |  |
| *lp_2858* |  | 1,79 | ABC transporter, ATP-binding protein,N-terminal domain | Multifunctional: Nucleotide transport and metabolism & Inorganic ion transport and metabolism |  |  |
| *lp_2870* | *-* | -2,17 | hypothetical protein |  | Intracellular | No pathway |
| *lp_2893* | *-* | 1,79 | ABC transporter ATP-binding and permease | Defense mechanisms | Multi-transmembrane | Sec-(SPI) |
| *lp_2948* | *-* | -1,86 | hypothetical protein | Function unknown | Intracellular /TMH start AFTER 60 | Possibly Sec- |
| *lp_2949* | *-* | -1,95 | hypothetical protein |  | Multi-transmembrane | Sec-(SPI) |
| *lp_2958* | *wapA* | -2,09 | wapA cell surface protein precursor, LPXTG-motif cell wall anchor | Cell wall/membrane/ envelope biogenesis | LPXTG motif Cell-wall anchored | Sec-(SPI) |
| *lp_2959* | - | -1,79 | transport protein | General functional prediction only | Multi-transmembrane | Sec-(SPI) |
| *lp_2960* | *-* | -2,10 | lipase/esterase, subfamily of SGNH-hydrolases |  | No information | No information |
| *lp_2964* | *-* | 1,86 | transcription regulator | Transcription | Intracellular | No pathway |
| *lp_2974* | *-* | -1,75 | ABC transporter substrate binding protein | General function prediction only | Lipid anchored | Sec-(SPII) |
| *lp_3000* | *-* | 1,64 | ABC transporter ATP-binding and permease | Defense mechanisms | Multi-transmembrane | Sec-(SPI) |
| *lp_3014* | *-* | -2,04 | extracellular protein | Cell wall/membrane/ envelope biogenesis | N-terminally anchored (No CS) | Sec-(SPI) |
| *lp_3015* | *-* | -1,77 | extracellular protein | Cell wall/membrane/ envelope biogenesis | Secretory(released) (with CS) | Sec-(SPI) |
| *lp_3049* | *-* | -1,79 | amino acid transport protein | Amino acid transport and metabolism | Multi-transmembrane | Sec-(SPI) |
| *lp_3128* | *-* | 1,66 | stress induced DNA binding protein | Inorganic ion transport and metabolism | Intracellular | No pathway |
| *lp_3139* | *-* | -1,55 | hypothetical protein |  | Multi-transmembrane | Sec-(SPI) |
| *lp_3165* | *-* | -1,65 | transposase, fragment |  | Intracellular | No pathway |
| *lp_3176* | *pkn2* | -1,66 | serine/threonine protein kinase | Multifunctional:  General function prediction only, Signal transduction mechanism, Transcription & Replication, recombination and repair | Multi-transmembrane | Sec-(SPI) |
| *lp_3180* | *-* | -1,70 | hypothetical protein |  | Multi-transmembrane | Sec-(SPI) |
| *lp_3185* | *-* | -2,43 | branched-chain amino acid transport protein | Amino acid transport and metabolism | Multi-transmembrane | Sec-(SPI) |
| *lp_3187* | *bar* | -1,53 | phosphinothricin N-acetyltransferase | Cell wall/membrane/ envelope biogenesis | Intracellular | No pathway |
| *lp_3190* | *hpk11* | 1,62 | histidine protein kinase; sensor protein | Signal transduction mechanism | Multi-transmembrane | Sec-(SPI) |
| *lp_3197* | *-* | 1,67 | hypothetical protein | Function unknown | Intracellular | No pathway |
| *lp_3204* | *nupC* | -1,64 | nucleoside transport protein | Nucleotide transport and metabolism | Multi-transmembrane | Sec-(SPI) |
| *lp_3205* | *-* | -1,94 | hypothetical protein | General function prediction only | N-terminally anchored (No CS) | Sec-(SPI) |
| *lp_3207* | *-* | -2,38 | PLP-dependent aminotransferase | Amino acid transport and metabolism & Transcription | Intracellular | No pathway |
| *lp_3214* | *-* | 1,62 | amino acid ABC transporter substrate binding protein | Multifunctional: Amino acid transport and metabolism & Signal transduction mechanism | Lipid anchored | Sec-(SPII) |
| *lp_3237* | *-* | 1,73 | HAD superfamily hydrolase | General function prediction only | Intracellular | No pathway |
| *lp_3245* | *-* | 1,58 | hypothetical protein | General function prediction only | Intracellular | No pathway |
| *lp_3256* | *-* | -3,25 | DegV family protein | Function unknown | Intracellular | No pathway |
| *lp_3322* | *-* | 1,64 | hypothetical protein |  | Intracellular | No pathway |
| *lp_3351* | *-* | 2,09 | hypothetical protein |  | Intracellular | No pathway |
| *lp_3356* | *-* | -1,70 | acetyltransferase | General function prediction only | Intracellular | No pathway |
| *lp_3358* | *-* | -1,77 | transport protein | General function prediction only | Multi-transmembrane | Sec-(SPI) |
| *lp_3368* | *-* | 2,96 | multidrug transport protein, major facilitator superfamily (MFS), EmrB/QacA subfamily, N-terminal domain | Inorganic ion transport and metabolism | No information | No information |
| *lp_3369* | *lmrB-C* | 2,74 | multidrug transport protein, major facilitator superfamily (MFS), EmrB/QacA subfamily, C-terminal domain |  | No infomation | No infomation |
| *lp_3374* | *1* | -1,57 | purine-cytosine transport protein | Nucleotide transport and metabolism | Multi-transmembrane(Lipid modified N-termini) | Sec-(SPII) |
| *lp_3377* | *-* | -1,65 | prophage Lp4 protein 13 |  | Multi-transmembrane | Sec-(SPI) |
| *lp_3380* | *-* | -2,01 | prophage Lp4 protein 10 |  | Intracellular | No pathway |
| *lp_3398* | *pacL3* | 2,77 | cation transporting P-type ATPase | Inorganic ion transport and metabolism | Multi-transmembrane | Sec-(SPI) |
| *lp_3459* | *-* | -1,73 | hypothetical protein |  | Intracellular | No pathway |
| *lp_3490* | *-* | 1,72 | FMN-binding protein | Multifunctional: Nucleotide transport and metabolism & Inorganic ion transport and metabolism | Intracellular | No pathway |
| *lp_3493* | *aroD* | -1,82 | 3-dehydroquinate dehydratase | Amino acid transport and metabolism | Intracellular | No pathway |
| *lp_3494* | *aroE* | -1,70 | shikimate 5-dehydrogenase | Amino acid transport and metabolism | Intracellular | No pathway |
| *lp_3501* | *-* | -1,63 | sugar phosphate isomerase/epimerase | Carbohydrate transport and metabolism | Intracellular | No pathway |
| *lp_3505* | *estA* | -1,67 | acetyl esterase (promiscuous) | General function prediction only | Intracellular | No pathway |
| *lp_3512* | *pbg8* | -1,68 | 6-phospho-beta-glucosidase | Nucleotide transport and metabolism & Inorganic ion transport and metabolism | Intracellular | No pathway |
| *lp_3513* | *pts30BCA* | -1,69 | beta-glucosides PTS, EIIBCA | Carbohydrate transport and metabolism | Multi-transmembrane | Sec-(SPI) |
| *lp_3514* | *bglG4* | -2,27 | transcription antiterminator, BlgB family | Transcription | Intracellular | No pathway |
| *lp_3531* |  | -1,66 | transcription regulator | Transcription | Intracellular | No pathway |
| *lp_3581* | *lamC* | -3,01 | two-component system histidine protein kinase; sensor protein | Multifunctional:  Signal transduction /  Inorganic ion transport and metabolism | Multi-transmembrane | Sec-(SPI) |
| *lp_3581a* | *lamD* | -4,58 | auto-inducing peptide (AIP) precursor (accesory gene regulator protein D, peptide pheromone precursor) |  | Intracellular | No pathway |
| *lp_3588* |  | 1,72 | Rrf2 family transcriptional regulator | Transcription | Intracellular | No pathway |
| *lp_3589* | *pox5* | 1,63 | pyruvate oxidase | Multifunctional:  Amino acid transport and metabolism, Coenzyme transport and metabolism, Nucleotide transport and metabolism & Inorganic ion transport and metabolism | Intracellular | No pathway |
| *lp_3658* | *rbsU* | 1,85 | ribose transport protein | Carbohydrate transport and metabolism, Energy production and conversion / Inorganic ion transport and metabolism | Multi-transmembrane | Sec-(SPI) |
| *lp_3659* | *rbsD* | 2,32 | D-ribose mutarotase | Multifunctional: Carbohydrate transport and metabolism / Energy production and conversion | Intracellular | No pathway |
| *lp_3660* | *rbsK1* | 1,66 | ribokinase | Multifunctional: Nucleotide transport and metabolism / Inorganic ion transport and metabolism | Intracellular | No pathway |
| *lp_tRNA21* |  | 1,74 |  |  |  |  |
| *lp_tRNA30* |  | 1,69 |  |  |  |  |
| *lp_tRNA44* |  | 1,78 |  |  |  |  |
| *lp_tRNA50* |  | 2,67 |  |  |  |  |

^a^ Fold change refers to growth in MRS supplemented with oleuropein 15 mM relative to growth in MRS without supplement

^b^ FDR ≤ 0.05

^c^ LocateP DataBase ([http://bamics2.cmbi.ru.nl/websoftware/locatep2/locatep2_start.php](http://bamics2.cmbi.ru.nl/websoftware/locatep2/locatep2_start.phpwww.cmbi.ru.nl/locatep-db/cgi-bin/locatepdb.py)) CS: CleavageSite; Sec-(SPI): Secretory Pathway I; Sec-(SPII): Secretory Pathway II.
